# Endocan removal during continuous renal replacement therapy: does it affect the reliability of this biomarker?

**DOI:** 10.1186/s13054-019-2469-7

**Published:** 2019-05-22

**Authors:** Patrick M. Honore, David De Bels, Rachid Attou, Sebastien Redant, Andrea Gallerani, Kianoush Kashani

**Affiliations:** 1 ICU Department, Centre Hospitalier Universitaire Brugmann, Place Van Gehuchtenplein,4, 1020 Brussels, Belgium; 2 Division of Nephrology and Hypertension, Division of Pulmonary and Critical Care Medicine, Mayo Clinic, Rochester, USA

We read the narrative review by De Freitas Caires et al. with great interest [1]. Acute kidney injury (AKI) is prevalent among patients with sepsis, and a substantial proportion of patients with sepsis-associated AKI (SA-AKI) require renal replacement therapy (RRT) [2]. Continuous RRT (CRRT) is increasingly used (~ 20% SA-AKI) among hemodynamically unstable septic shock patients [2]. Endocan as a novel endothelium-derived soluble dermatan sulfate proteoglycan has a molecular mass of around 20 kDa [3]. The contemporary CRRT membranes are able to remove molecules as large as 35 kDa. Hence, endocan could be removed by CRRT [4]. When new highly adsorptive membranes (HAM) with high absorptive abilities are used, the ability of CRRT to eliminate endocan could be even enhanced [4]. Therefore, the reliability of endocan during CRRT could be altered. De Freitas Caires et al. show that endocan appeared as a consistent good diagnostic criterion as well as procalcitonin (PCT) and could potentially be used for de-escalation therapy in the future (requiring new studies obviously) as PCT. Accordingly (if endocan is used for de-escalation in the future), falsely low endocan in CRRT patients, in turn, could lead to an earlier de-escalation of antibiotics and level of care for septic patients. There has been no investigation on the performance of endocan on patients who receive CRRT. Therefore, we believe there is a critical need for a future study with a focus on the performance of the currently known sepsis biomarkers among those who receive CRRT [5].

## Abbreviations

AKI: Acute kidney injury
CRRT: Continuous renal replacement therapy
HAM: Highly adsorptive membranes
PCT: Procalcitonin
RRT: Renal replacement therapy
SA-AKI: Sepsis-associated AKI
